# Psychotherapy of trauma and resources of the patient: theoretical and practical aspects in the psychotherapeutic practice

**DOI:** 10.3402/ejpt.v4i0.22984

**Published:** 2013-12-06

## XIII ESTSS Conference: “Trauma and its clinical pathways: PTSD and beyond”, Bologna, June 2013 ORAL, JUNE 8 HALL SYDNEY

## Open Papers: Approcci di cura e trauma

### Psicoterapia del trauma e risorse del paziente: aspetti teorici e pratici nella prassi psicoterapica 17.30–17.45

#### Marialfonsa Fontana Sartorio Associazione Qualità e Formazione, Milan - Sipst, Milan, Italy

Poiché sappiamo dai più recenti studi delle neuroscienze, della psicologia e della psicoterapia che ogni crisi o conflitto sufficientemente elaborato viene memorizzato nel nostro cervello come nuova esperienza che modifica le nostre cognizioni e i nostri comportamenti, si è posto il problema nella psicoterapia del trauma di come facilitare questo processo e come rinforzare le competenze specifiche del paziente di fronte ad ansia e traumatizzazione.

Centrale si è dimostrato il lavoro sulla attivazione e rinforzo delle risorse personali, sia nell'elaborazione del trauma, sia a scopo preventivo, in quanto vissuti di estrema impotenza attivano stati dell'Io connessi a sentimenti di inermità e disperazione, poichè l'esperienza traumatica intacca l'immagine positiva di sè.

Da ciò derivano restringimento della percezione e impossibilità di valutazione, il cervello non riesce più a trovare soluzioni creative. Una situazione che si consolida in presenza di PTSD, in cui viene bloccato il processo di elaborazione dell'evento vissuto, incistendolo in un perenne presente, diventando così di ostacolo al dispiegarsi libero della creatività dell'individuo.

A tale scopo si menzionano interventi ormai consolidati, come il Psychoenergetic Drawing^®^ (www.qualitaeformazione.com) che si basa sull'aspetto positivo e progettuale insito nel concetto di complesso di Jung, e il TRUST-Resilienz-Training/TRUST-RT^®^ di Christa Diegelmann e di Margarete Isermann (www.idinstitut.de) riconosciuto come corso formativo presso la Deutsche Psychologen Akademie dal 2012, atte a rinforzare la resilienza psichica.

Non si tratta di rincorrere un ipotetico concetto di “sanità”, bensì di distinguere tra benessere e crescita postraumatica, cioè quell'insieme di cambiamenti positivi che sono il risultato dell'elaborazione dell'evento traumatico, sostenendo quindi la fiducia nelle potenzialità dell'individuo a integrare il trauma nel proprio mondo e ritrovare un più complesso equilibrio per continuare il personale processo di individuazione.

### Psychotherapy of trauma and resources of the patient: theoretical and practical aspects in the psychotherapeutic practice

From the most recent studies in neuroscience, psychology, and Psychotherapy, it is known that any elaborated crisis or conflict gets registered in our brain as a new experience, and changes our knowledge and behaviour. We addressed this issue in the psychotherapy of trauma and also the ways to facilitate this process and strengthen the patient's specific competencies dealing with anxiety and trauma.

It is very important to work on the activation and reinforcement of personal resources, both in the elaboration of trauma and as a preventive measure, because extreme impotence activates ego states related to feelings of helplessness and despair, as the traumatic experience undermines the positive image of ourselves.

This leads to narrowing of perception and consequently inability to evaluation. The brain will no longer be able to find creative solutions: a situation that is consolidated in the presence of PTSD, when the process of elaborating experiences is blocked, frozen in a perpetual present causing an obstacle to the free unfolding of the individual creativeness.

For this purpose, we mentioned consolidated intervention tools: Psychoenergetic Drawing^®^ (www.qualitaeformazione.com), which is based on the positive and projecting aspect inherent in the concept of complex of Jung, and the TRUST-Resilienz-Training/TRUST-RT^®^ of Christa Diegelmann and Margarete Isermann (www.idinstitut.de) recognized as formative course at the Deutsche Psychologen Akademie from 2012, both designed to strengthen the psychic resilience.

It does not deal with a hypothetical concept of ‘health’; on the contrary, it deals with the ways to distinguish well-being and post-traumatic growth, that is to say, all those positive changes that result from the processing of the traumatic event, trusting thereby in the potentialities of individuals to integrate trauma in their own world and to find a more complex balance to continue their process of personal identification.

